# Digital Gaming for Nutritional Education: A Survey on Preferences, Motives, and Needs of Children and Adolescents

**DOI:** 10.2196/10284

**Published:** 2019-02-13

**Authors:** Sophie Laura Holzmann, Felicitas Dischl, Hanna Schäfer, Georg Groh, Hans Hauner, Christina Holzapfel

**Affiliations:** 1 Institute for Nutritional Medicine Else Kroener-Fresenius-Center for Nutritional Medicine University Hospital “Klinikum rechts der Isar”, Technical University of Munich Munich Germany; 2 Research Group Social Computing Department of Informatics Technical University of Munich Garching Germany; 3 Nutritional Medicine Unit Institute for Food and Health Technical University of Munich Freising Germany

**Keywords:** adolescents, children, communication, motives, mobile phone, needs, nutrition, obesity, overweight, preferences, serious games, survey

## Abstract

**Background:**

Use of novel information and communication technologies are frequently discussed as promising tools to prevent and treat overweight and obesity in children and adolescents.

**Objective:**

This survey aims to describe the preferences, motives, and needs of children and adolescents regarding nutrition and digital games.

**Methods:**

We conducted a survey in 6 secondary schools in the southern region of Germany using a 43-item questionnaire. Questions referred to preferences, motives, and needs of children and adolescents regarding nutrition and digital games. In addition, knowledge regarding nutrition was assessed with 4 questions. We collected self-reported sociodemographic and anthropometric data. Descriptive statistical analyses were performed using SPSS.

**Results:**

In total, 293 children and adolescents participated in the study, with ages 12-18 years (137 girls, 46.8%), weight 30.0-120.0 (mean 60.2 [SD 13.2]) kg, and height 1.4-2.0 (mean 1.7 [SD 0.1]) m. A total of 5.5% (16/290) correctly answered the 4 questions regarding nutrition knowledge. Study participants acquired digital nutritional information primarily from the internet (166/291, 57.0%) and television (97/291, 33.3%), while school education (161/291, 55.3%) and parents or other adults (209/291, 71.8%) were the most relevant nondigital information sources. Most participants (242/283, 85.5%) reported that they regularly play digital games. More than half (144/236, 61.0%) stated that they play digital games on a daily basis on their smartphones or tablets, and almost 70% (151/282, 66.5%) reported playing digital games for ≤30 minutes without any interruption. One-half of respondents (144/280, 51.4%) also stated that they were interested in receiving information about nutrition while playing digital games.

**Conclusions:**

This survey suggests that nutrition knowledge in children and adolescents might be deficient. Most children and adolescents play digital games and express interest in acquiring nutritional information during digital gameplay. A digital game with a focus on sound nutrition could be a potential educational tool for imparting nutrition knowledge and promoting healthier nutrition behaviors in children and adolescents.

## Introduction

### Overweight and Obesity in Children and Adolescents

Overweight and obesity are major public health concerns worldwide [1]. In Germany, 15% of children and adolescents aged 3-17 years are overweight, while 6.3% are obese [2]. The number of children and adolescents with obesity, aged 5-19 years, has increased in most regions and countries from 1975 to 2016 [3]. The major cause of overweight and obesity is an energy imbalance between caloric intake and energy expenditure. An obesogenic environment, dominated by a Western diet high in energy-dense foods, is linked to obesity [1]. In addition, there is a growing trend toward a sedentary lifestyle [1,4]. Overweight and obesity require effective prevention and treatment strategies, especially in children and youths. At present, there is growing interest in exploring the potential of new information and communication technologies (ICTs), for example, mobile apps, for tackling the obesity epidemic. These novel tools are low cost, ubiquitous, and almost unlimitedly scalable. Studies have already demonstrated that young people prefer health information through ICTs rather than through printed materials [5,6].

### Prevention and Treatment Strategies—Serious Games

A novel ICT approach is “serious games,” which is a term used for digital games that are designed for educational and training purposes [7]. Serious gaming is a promising method to convey health-related information and promote change in health-related behaviors because of its exciting, enjoyable, and intrinsically motivational nature [8-13]. Several studies have investigated the effects of serious games on health-related outcomes like nutrition knowledge, dietary behavior, physical activity, and body mass index [14-20]. A randomized controlled trial observed that 10-12-year-old children who played video games significantly increased fruits and vegetable consumption compared with the control group, who played serious games on websites [21]. A systematic review showed that most studies achieved positive results using video games in the prevention and treatment of obesity in children and adolescents. The authors emphasized that video games provide an additional and supporting element in preventing and treating obesity along with the potential to increase treatment compliance and to promote success [22]. The “Alien Health Game,” a nutrition instruction game, increased short-term nutrition knowledge among primary school children [13]. Owing to the high acceptance and widespread usage of digital games, children and adolescents seem to be a suitable target group for the serious gaming approach. Nevertheless, exposure to electronic gameplay may be associated with overweight and obesity because of its potential to increase sedentary behavior and consumption of energy-dense foods and beverages [23,24]. In addition, general and especially excessive use of digital devices is associated with negative effects such as school difficulties, sleep disturbance, depression, and Attention-Deficit/Hyperactivity Disorder [24-26]. Nowadays, serious and exergames are developed to overcome the adverse effects of gameplay by education combined with entertainment, known as “edutainment” [11,27]. Serious games may enable health educators to integrate health education into already existing game-based activities.

### Aim

The objective of this survey was to gather information about the preferences, motives, and needs of children and adolescents regarding nutrition and digital games. The results of this survey are intended to be used for the development of a serious nutrition game tailored to children and adolescents.

## Methods

### Design and Protocol

The Ethical Committee of the Faculty of Medicine at the Technical University of Munich and the Rosenheim School Board approved this survey, which was conducted in 6 secondary schools in the city and administrative district of Rosenheim (Bavaria, Germany) between June 2016 and July 2017. We obtained written consent from parents or legal guardians of each participant.

### Recruitment and Procedure

The Rosenheim School Board invited 23 secondary schools to participate in the survey. Children and adolescents were recruited by school teachers. We conducted the survey under standardized conditions according to a detailed survey protocol, which was executed by the study team. Each participant received a hard copy of the questionnaire. The study team read the introduction aloud, after which participants completed the questionnaire on their own. Children and adolescents who were not given parental permission to participate received a nutrition quiz. The maximum time for completing the questionnaire was 45 minutes, which corresponds to 1 classroom hour.

### Questionnaire: Development and Design

An interdisciplinary team of nutritionists, economists, sociologists, and computer scientists developed a target group-specific questionnaire. The preliminary questionnaire was pretested for ease-of-use and understandability by a subset of 26 individuals aged 14-17 years. The final questionnaire was designed using EvaSys V7.0 (2101). The introduction included information regarding the research project, data protection, voluntary participation, and contained instructions for completing the questionnaire. The main part consisted of questions referring to 3 different subjects—nutrition (16 questions), digital games (21 questions), and personal characteristics (6 questions). The nutrition questions referred to nutritional communication, behavior, and knowledge and focused on preferences, motives, and needs. The area on digital games consisted of questions on preferences, motives, and needs regarding gaming behavior in general and digital gaming behavior in particular. To design a target group-tailored serious game, questions concerning the design of a digital game were also asked. In addition, we obtained sociodemographic (age, sex, and home environment) and self-reported anthropometric data (weight and height). The mean value for weight and height was calculated for participants who provided ranges for these values. The literature indicates that self-reporting of weight and height is an appropriate and reliable assessment method among adolescents and young adults compared with measured anthropometric data [28,29]. To maintain the attention and motivation of respondents, introductory questions were used. In addition, the filter question “Do you play digital games (smartphone, computer, console, apps)?” was asked to ensure that subsequent questions were answered only by participants who actually play digital games. The majority of the questions were closed and semiended with single or multiple-choice answer options; however, the questionnaire also contained a few open-ended questions. The questions were short, unidimensional, and easy to understand. To avoid influencing the response behavior, questions were designed to be neutral and target group-specific. Furthermore, a “do not know” or “no answer applies” option was mostly provided to prevent incorrect responses and remove pressure. Furthermore, questions were formulated in a way to avoid social desirability.

### Statistical Analyses

Data were initially checked for integrity and plausibility. Respondents with ≥8 unanswered or invalid questions were excluded. Therefore, the number of respondents varied from question to question. Furthermore, the statistical analysis was focused on selected questions, which are directly associated with the game development. In total, 21 questions were considered for the underlying analysis (Multimedia Appendix 1). The descriptive data analysis (frequencies and percentages) was performed using Microsoft Excel 2013 (Microsoft Corp) and SPSS version 24 (IBM Corp).

## Results

### Participants’ Characteristics

In total, 293 German-speaking children and adolescents (137 girls and 156 boys) from the 7th to 8th grade who were not affected by writing disabilities participated in this study. Table 1 presents participants’ demographic and anthropometric characteristics. Children and adolescents (137/293, 46.8% girls) aged, on average, 14.7 (SD 1.2) years. Weight ranged 30.0-120.0 (mean 60.2 [SD 13.2]) kg, while height varied between 1.4 m and 2.0 (mean 1.7 [SD 0.1]) m. Most respondents (278/293, 94.9%) reported that they live “at home.”

### Digital and Nondigital Sources of Nutritional Information

Table 2 shows sources of digital and nondigital nutritional information that are currently used, as well as additionally desired by respondents. More than one-half of the participants (166/291, 57.0%) reported using the internet as a digital information source. In total, 71.8% (209/291) participants indicated obtaining nondigital nutritional information from parents and adults in general. In contrast, most participants wanted to receive additional nutritional information through apps (49/274, 17.9%) and school education (108/274, 39.4%). Fewer than 10% (23/291, 7.9%) of participants reported currently using or wanting to use additionally (19/274, 6.9%) digital games to familiarize themselves with nutrition knowledge. Twice as many boys (16/156, 10.3%) as girls (7/137, 5.1%) used digital games for nutritional information; however, this gender-specific difference was not observed for any other currently used information source.

**Table 1:** Participants’ characteristics (N=293).

Variable		Participants, n (%)		Participants, mean (SD)		Minimum, maximum	
**Age (years)^a^**							
	12-14		148 (50.5)		N/A^b^		N/A
	15-17		134 (45.7)		N/A		N/A
	18		11 (3.8)		N/A		N/A
**Sex**							
	Girls		137 (46.8)		N/A		N/A
	Boys		156 (53.2)		N/A		N/A
**Anthropometric data**							
	Weight (kg)		276 (94.2)		60.2 (13.2)		30.0, 120.0
	Height (m)		289 (98.6)		1.7 (0.1)		1.4, 2.0
**Home environment**							
	At home		278 (94.9)		N/A		N/A
	Alone		2 (0.7)		N/A		N/A
	Other		13 (4.4)		N/A		N/A

^a^Participants: mean 14.7 (SD 1.2); minimum 12, maximum 18.

^b^N/A: not applicable.

**Table 2:** Sources of nutritional information.

Nutritional information source^a^			All, n (%)		Girls, n (%)		Boys, n (%)	
**Currently used^b^**								
	**Digital**							
		Television		97 (33.3)		39 (28.7)		58 (37.4)
		Internet		166 (57.0)		79 (58.1)		87 (56.1)
		Social networks		78 (26.8)		42 (30.9)		36 (23.2)
		Apps		33 (11.3)		15 (10.9)		18 (11.5)
		Digital games		23 (7.9)		7 (5.1)		16 (10.3)
	**Nondigital**							
		School		161 (55.3)		76 (55.5)		85 (54.5)
		Parents and adults		209 (71.8)		100 (73.0)		109 (69.9)
		Friends		54 (18.6)		32 (23.4)		22 (14.1)
		Books & newspapers		74 (25.4)		44 (32.1)		30 (19.2)
	**Other**							
		Unconscious		51 (17.5)		19 (13.9)		32 (20.5)
		No answer applies		13 (4.5)		4 (2.9)		9 (5.8)
**Additionally desired^c^**								
	**Digital**							
		Television		45 (16.4)		19 (14.5)		26 (18.2)
		Internet		46 (16.8)		22 (16.8)		24 (16.8)
		Social networks		34 (12.4)		15 (11.5)		19 (13.3)
		Apps		49 (17.9)		29 (22.1)		20 (14.0)
		Digital games		19 (6.9)		10 (7.6)		9 (6.3)
	**Nondigital**							
		School		108 (39.4)		43 (32.8)		65 (45.5)
		Parents & adults		46 (16.8)		23 (17.6)		23 (16.1)
		Friends		17 (6.2)		12 (9.2)		5 (3.5)
		Books & newspapers		31 (11.3)		22 (16.8)		9 (6.3)
	**Other**							
		Unconscious		29 (10.6)		15 (11.5)		14 (9.8)
		No answer applies		74 (27.0)		30 (22.9)		44 (30.8)

^a^Multiple responses allowed.

^b^Where are you informed about nutrition?/Where do you inform yourself about nutrition?; n (%)=291 (100).

^c^Where do you want to be additionally informed about nutrition?; n (%)=274 (100).

### Knowledge About Nutrition

In total, 4 questions were asked to evaluate the participants’ nutrition knowledge (Table 3). Less than one-fourth of respondents (66/290, 22.8%) answered the question regarding the daily recommended fruits and vegetable consumption according to the German Nutrition Society (DGE) correctly. The common response to the question “Do you know what a food pyramid is?” was “Yes” (260/293, 88.7%). Almost three-fourths (210/291, 72.2%) of the survey population correctly indicated that fish should be eaten once or twice per week. In total, 24.7% (72/291) participants responded incorrectly regarding the calorie content of 100 g of sugar; almost one-half of children and adolescents (145/291, 49.8%) replied with “I don’t know.” Moreover, 10.3% (30/290) and 18.2% (53/291) reported not knowing the correct answer regarding recommendations of daily fruits and vegetable or fish consumption. Overall, only 5.5% (16/290) of participants correctly answered all 4 questions regarding nutrition knowledge.

**Table 3:** Nutrition knowledge in children and adolescents.

Question^a^	n (%)	Correct answer			Incorrect answer				Answer “I don’t know”			
		All, n (%)	Girls, n (%)	Boys, n (%)		All, n (%)	Girls, n (%)	Boys, n (%)		All, n (%)	Girls, n (%)	Boys, n (%)
How many portions of fruits and vegetables should be eaten (portion = a handful) each day?	290 (100)	66 (22.8)	31 (23.1)	35 (22.4)		194 (66.9)	87 (64.9)	107 (68.6)		30 (10.3)	16 (11.9)	14 (9.0)
Do you know what a food pyramid is?	293 (100)	260 (88.7)	129 (94.2)	131 (84.0)		33 (11.3)	8 (5.8)	25 (16.0)		N/A^b^	N/A	N/A
How often should fish be eaten each week?	291 (100)	210 (72.2)	101 (73.7)	109 (70.8)		28 (9.6)	11 (8.0)	17 (11.0)		53 (18.2)	25 (18.3)	28 (18.2)
How many calories [kcal] are in 100g of sugar?	291 (100)	74 (25.4)	24 (17.7)	50 (32.3)		72 (24.7)	34 (25.0)	38 (24.5)		145 (49.8)	78 (57.4)	67 (43.2)

^a^Single response allowed.

^b^N/A: not applicable.

### Digital Gameplay: Preferences, Motives, and Needs

Multimedia Appendix 2 presents motives, behavior, and preferences regarding digital games. More than 80% (242/283, 85.5%) of the survey population reported playing digital games. Children and adolescents primarily play digital games if they were in the mood (209/282, 74.1%) or if they are bored (164/282, 58.2%), while 8.5% (24/282) reported happiness or sadness (15/282, 5.3%) as motivators for digital gameplay. More boys than girls indicated playing digital games when they felt like gaming or when they were happy. Children and adolescents often play digital games if they were alone at home (117/282, 41.5%) or stay with friends (54/282, 19.2%), while boys reported this behavior 2-3 times more often than girls. Playing time was observed to be dependent on the digital device. While most participants play digital games continuously for up to 30 minutes on a smartphone or tablet (151/227, 66.5%), less than half (57/209, 27.3%) play for up to 30 minutes on a personal computer (PC) or console. Participants reported playing digital games ≥1 hour on PC or consoles (116/209, 55.5%), which was 3 times more often than those playing digital games for the same amount of time on smartphones or tablets (41/227, 18.1%). Compared with female participants (6/79, 7.6%), male children and adolescents were far more likely to play digital games for >1 hour (47/130, 36.2%). A similar trend could be detected for a gaming duration lasting 1 hour. The most common answer regarding gaming frequency on any device was daily, with differences observed between girls and boys. In addition to the motives behind digital gameplay and the behavior itself, the questionnaire also asked about gaming preferences. More than one-half of the survey population preferred that teammates in a digital nutrition game are friends (191/286, 66.8%) or individuals with similarities, such as hobbies or eating behaviors (203/286, 71.0%); however, some participants 7.4% (21/286) favored playing without a team. Almost one-half of children and adolescents reported enjoying digital gameplay with ≤5 players (116/255, 45.5%), and 22.8% (58/255) of participants liked playing alone. Close to one-half of participants (119/277, 43.0%) preferred an older or same-aged human as a game character, while 19.1% (53/277) preferred a fantasy character. The main proportion of the survey population reported that they prefer learning about nutrition through a quiz (157/287, 54.7%) or by solving tasks (123/287, 42.9%). Some participants 12.9% (37/287) did not want to learn anything in a digital nutrition game, with boys about 3 times more likely to report this answer (29/151, 19.2%) than girls (8/136, 5.9%).

## Discussion

### Nutrition Knowledge in Children and Adolescents

This survey suggests that nutrition knowledge in children and adolescents may be limited. Only 6% (16/290) correctly answered the 4 questions regarding nutrition knowledge. Moreover, one-fourth (66/290, 22.8%) of participants knew that eating fruits and vegetables 5 times per day is recommended, and nearly the same number (74/291, 25.4%) correctly answered questions about the calorie content of sugar. These findings are consistent with the results of a recent study by the German Nutrition Society, which indicated that around 50% of German adolescents are inadequately informed about the components of a healthy diet [30]. The National Pupil Survey (2013) revealed that only one-half of children and adolescents aged 11-16 years correctly answered a question regarding the recommendation for daily fish intake [31]. This survey indicates that >70% of participants knew that fish should be eaten once or twice per week. As evidenced by the National Pupil Survey, 88% of adolescents know that 5 portions of fruits and vegetables should be eaten each day [31]. Epidemiological data have demonstrated that knowledge about nutritional recommendations as well as the intake of fruits and vegetables has to be improved in German adolescents. A German survey revealed that only 25%-29% of girls and 16%-18% of boys met the fruits or vegetable intake recommendation [32]. It should be mentioned that this survey provides no representative data of nutrition knowledge among children and adolescents. Furthermore, it cannot be ruled out that respondents affirmed the question without even having knowledge of it. Hence, the 4 questions about nutrition knowledge can only provide a small impression about respondents’ nutrition knowledge. In addition, it is noteworthy that many factors have an impact on children’s and adolescents’ nutrition knowledge. According to this survey, the majority of children and adolescents (72%) consult their parents or adults regarding nutrition. Wansink stated that a “home’s nutritional gatekeeper is the biggest food influence in the nutrition life” of all family members [33]. The findings of Qian et al showed that children with low parent education level tend to have a less comprehensive nutrition knowledge than children from parents who are more highly educated [34]. As the survey was only conducted in secondary schools, it could be assumed that the education level of parents might have been rather low. Further literature indicates that nutritional education of parents can be effective in improving children’s diet [35,36]. A recent systematic review of parent-targeted, in-home interventions resulted in a small but significant increase in fruits consumption in children [35]. These facts need to be considered, as nearly 95% of children and adolescents in this survey lived at home. Findings regarding the effectiveness of nutritional education programs on dietary knowledge and food behaviors are controversial [37]. A study showed that students aged 14-19 years significantly improved their nutrition knowledge after attending a nutritional course at school [38]. A 5-day physical activity and nutritional intervention program in children with overweight and obesity resulted in higher rates of physical activity, as well as in a reduction of consumed sugar and sweets [39]. Moreover, reviews demonstrated that nutritional education could be effective in improving healthy eating behaviors [40]; in contrast, a cross-sectional survey revealed that the “knowledge of healthy foods does not translate to healthy snack consumption” [41]. By improving nutrition knowledge and health attitudes children and adolescents may develop a healthier lifestyle and, thus, would be at lower risk of becoming overweight or obese [38].

### Implications for Game Design

This study shows that more than one-half of children and adolescents would like to receive nutritional information through digital games. The majority stated that they would like to learn about nutrition in a digital game by playing a quiz (55%) or by solving tasks (43%). Therefore, different types of quizzes should be considered in the development of a nutrition game [42]. A recent study in children investigated the delivery of nutritional information through a gaming app and demonstrated that learning of nutritional information was improved with repeated exposure [43]. To convey knowledge effectively through a game, different elements should be considered in the game design [42]. Many participants (43%) reported preferring a fantasy character as a game character. These findings are similar to the results of a survey about serious games conducted among 465 Asian pharmacy students; most participants (60%) preferred a fantasy, medieval, and mythical setting, while 41% wanted an adventure storyline [44]. For example, the “Move2Play” game contains an avatar which can be personalized by users. A small study showed that adolescents enjoyed customizing their avatar [45]. It is also worth mentioning that children and adolescents in this survey preferred playing digital games with friends (191/286, 66.8%) and only one-fifth of participants (58/255, 22.8%) played digital games by themselves. The literature confirms that digital gaming has a great social value. In a survey on video gameplay among 1254 US 7th- and 8th-grade students, only 18% of male and 12% of female children and adolescents indicated that they always play alone [46]. In addition, digital gaming as a social activity was confirmed in a focus group of 42 male adolescents [46]. Considering these findings, players should be given an opportunity to create social groups in a game [42]. Furthermore, social components, such as chat interaction, group profiles, and social games or challenges, should be implemented [42].

### Digital Gameplay—Preferences, Motives, and Needs

This survey data show that >85% of children and adolescents play digital games. According to the survey by Olson et al, only 6% of children and adolescents did not play any digital games in the 6 months prior to the survey [46]; these results confirm that children and adolescents are frequent users of digital devices [47]. Adolescents aged 15-18 years spend on average 22 minutes per day on video gaming on cell phones, and 31 minutes per day on consoles [47]. Furthermore, a German survey showed that two-thirds of participants aged 12-19 years (n=1200) played digital games (smartphone, PC [offline], console, online, and tablet) regularly (daily or several times a week), whereas only 8% never played digital games [48]. Regarding the frequency of digital gameplay, these results are similar to the current findings. Almost two-thirds of participants specified playing digital games daily or weekly on smartphones or tablets, and more than two-thirds played on a PC or console. The use of educational games as a viable teaching strategy can promote enjoyment and, therefore, may enhance the retention of information in the long-term [12]. In contrast, excessive use of digital games can generate negative health effects in children and adolescents [23]. Consequently, there is an ongoing debate as to whether electronic gameplay is a “health hazard” or a “health promoter” [49]. The question of why young people play digital games is also addressed in this survey. Most children and adolescents have an emotionally induced digital gameplay experience and primarily replied to the question “When do you often play digital games?” with “pleasure” and “boredom.” Olson et al showed that 45% of male and 29% of female participants said that they played to “To get my anger out,” while 25% of boys and 11% of girls selected the answer “cope with anger” [46,50]. Negative emotions, such as “sadness,” were only reported by around 5% of young people in this survey, whereas the answer option “anger” was not present within this questionnaire. The fact that most young people play digital games when they are in a positive mood could be exploited to transfer knowledge playfully and entertainingly through digital games.

### Serious Games—Serious Design

Although there are a growing market supply and demand for health-related games, little is known about game design aspects and preferences, motives, and needs of children and adolescents regarding digital nutrition games [51]. It is important to emphasize that qualitative research (eg, interviews) prior to game design is critical [52]. Consideration of the “needs, interests, perspectives, and preferences” of the target group in the development of health games may result in better targeted games [52]. Furthermore, collaborations with health professionals from an early design stage are necessary, both to ensure that the content is accurate and to have the game validated from a clinical viewpoint. The target group needs to be involved, especially to improve the usability. It is well known that health promotion and disease prevention programs suffer from poor adherence and compliance by participants, often because they are designed without addressing the target group [14,53]. This also applies to the field of health games. In their recently published review, Lu and Kharrazi examined almost 2000 health-related games (from 1983 to 2016) for usability and concluded that one of the main limitations is the lack in customization and feedback [54].

### Digital Games for Nutritional Education at Schools

Furthermore, it has to be noted that most children and adolescents like to receive additional nutritional information through apps or at school. According to a study of 505 American teachers, more than one-half (55%) reported using games as an educational element in school teaching [55]. The advantage of serious games is to educate the target group through entertaining, enjoyable, and intrinsically motivating game elements and experiences [11,27]. Therefore, a serious nutrition game may be a suitable, educative method to impart nutrition knowledge and to promote healthy dietary behaviors in children and adolescents.

### Limitations

This survey revealed findings about digital nutrition games among children and adolescents, yet it has some limitations. The survey was conducted in one school area and type. Therefore, it is limited by sample size and homogeneity. Moreover, present findings are not representative, especially concerning nutrition knowledge, which was assessed on the basis of 4 questions. Furthermore, it cannot be ruled out that the participation rate, which cannot be calculated owing to missing data (invited vs participated children and adolescents), is biased by the motivation and engagement of teachers, parents, and participants themselves. The nonvalidated questionnaire used in the survey was internally generated to obtain all the relevant information for a target group-specific game design. As the age of participants is only available as life years without months, no appropriate weight classification for children and adolescents (percentiles/z-scores) could be calculated. The results are further limited owing to missing information about parental education level. Future research should address more diverse survey populations across entire Germany, providing further insights and expanding the generalizability.

### Conclusions

Results revealed that children and adolescents are interested in nutritional information. Present findings support previous surveys that there is a lack of nutrition knowledge in this target group. Children and adolescents stated a preference for apps and school education in order to receive additional nutritional information. Furthermore, the target group was interested in digital games as well. Therefore, such preferences and interests could be implemented in an educational app, for example, for schools to increase nutrition knowledge and to improve dietary behavior.

## Appendix

Multimedia Appendix 1 Questionnaire excerpt from Holzmann et al (Digital Gaming for Nutritional Education).

Multimedia Appendix 2 Table from Holzmann et al (Digital Gameplay: Motives, Behavior, and Preferences).

## Abbreviations

ICT: information and communication technology
PC: personal computer
