# Supplementary material for: Digital Gaming for Nutritional Education: A Survey on Preferences, Motives, and Needs of Children and Adolescents
Source: JMIR Form Res. 2019 Feb 13;3(1):e10284. doi: 10.2196/10284 (PMC6391648; doi:10.2196/10284)
Supplement: Multimedia Appendix 1 [file formative_v3i1e10284_app1.pdf]

Multimedia Appendix 1. Questionnaire Excerpt – Holzmann *et al*.: Digital Gaming for Nutritional Education

This is an excerpt of the German questionnaire applied in the survey. In the following some questions are listed in German language. The questions are mentioned in English in the manuscript.

Table 2

1. Wo wirst Du über Ernährung informiert / Wo informierst Du Dich über Ernährung?
(Kreuze bitte alle passenden Antworten an!)

- ☐ Schulunterricht
- ☐ Eltern / Erwachsene
- ☐ Freunde/innen
- ☐ Bücher / Zeitschriften
- ☐ Unbewusst (Werbung)
- ☐ Fernsehen
- ☐ Internet
- ☐ Soziale Netzwerke
- ☐ Apps
- ☐ Digitale Spiele (Smartphone, PC, Konsole)
- ☐ Keine Antwort trifft zu

2. Wo willst Du zusätzlich über Ernährung informiert werden?
(Kreuze bitte alle passenden Antworten an!)

- ☐ Schulunterricht
- ☐ Eltern / Erwachsene
- ☐ Freunde/innen
- ☐ Bücher / Zeitschriften
- ☐ Unbewusst (Werbung)
- ☐ Fernsehen
- ☐ Internet
- ☐ Soziale Netzwerke
- ☐ Apps
- ☐ Digitale Spiele (Smartphone, PC, Konsole)
- ☐ Keine Antwort trifft zu

Table 3

3. Wie viele Portionen Obst und Gemüse sollte man täglich essen? (Portion = eine Handvoll)
(Kreuze bitte nur eine Antwort an!)

- ☐ 0 Portionen
- ☐ 1-2 Portionen
- ☐ 5 Portionen
- ☐ So viele Portionen wie man möchte
- ☐ Weiß nicht

4. Weißt Du, was eine Lebensmittelpyramide ist?
(Kreuze bitte nur eine Antwort an!)

- ☐ Ja
- ☐ Nein

5. Wie oft sollte man Fisch pro Woche essen?
(Kreuze bitte nur eine Antwort an!)

- ☐ Jeden Tag
- ☐ 1-2 Mal pro Woche
- ☐ 3-4 Mal pro Woche
- ☐ Weiß nicht

6. **Wie viele Kalorien [kcal] enthalten 100 g Zucker?**
   (Kreuze bitte nur eine Antwort an!)
- ☐ 306 kcal
- ☐ 406 kcal
- ☐ 506 kcal
- ☐ Weiß nicht

Multimedia Appendix 2

7. **Wann spielst Du häufig digitale Spiele?**
   (Kreuze bitte alle passenden Antworten an!)
   Wenn ich ...
- ☐ Lust habe
- ☐ fröhlich bin
- ☐ traurig bin
- ☐ gelangweilt bin
- ☐ bei Freunden/innen bin
- ☐ alleine zu Hause bin
- ☐ unterwegs bin
- ☐ in der Schule bin
- ☐ Andere Situation:_________________________
- ☐ Ich spiele nicht
- ☐ Weiß nicht

8. **Spielst Du digitale Spiele (Smartphone, PC, Konsole, Apps)?**
   (Kreuze bitte nur eine Antwort an!)
- ☐ Ja
- ☐ Nein

9. **Wie lange spielst Du ohne Unterbrechung digitale Spiele auf dem Smartphone / Tablet / Apps?**
   (Kreuze bitte nur eine Antwort an!)
- ☐ < 5 Minuten
- ☐ ~ 5 Minuten
- ☐ ~ 15 Minuten
- ☐ ~ 30 Minuten
- ☐ ~ 60 Minuten
- ☐ > 60 Minuten
- ☐ Ich spiele nicht
- ☐ Weiß nicht

10. **Wie lange spielst Du ohne Unterbrechung digitale Spiele am PC / auf der Konsole?**
    (Kreuze bitte nur eine Antwort an!)
- ☐ < 5 Minuten
- ☐ ~ 5 Minuten
- ☐ ~ 15 Minuten
- ☐ ~ 30 Minuten
- ☐ ~ 60 Minuten
- ☐ > 60 Minuten
- ☐ Ich spiele nicht
- ☐ Weiß nicht

11. **Wie oft spielst Du digitale Spiele auf dem Smartphone / Tablett / Apps?**
    (Kreuze bitte nur eine Antwort an!)
- ☐ 1-2 Mal / Tag
- ☐ 3-5 Mal / Tag
- ☐ 6-10 Mal / Tag
- ☐ Mehr als 10 Mal / Tag
- ☐ Mehrmals in der Woche
- ☐ Mehrmals im Monat

- ☐ Ich spiele nicht
- ☐ Weiß nicht

12. Wie oft spielst Du digitale Spiele am PC / auf der Konsole?
(Kreuze bitte nur eine Antwort an!)

- ☐ 1-2 Mal / Tag
- ☐ 3-5 Mal / Tag
- ☐ 6-10 Mal / Tag
- ☐ Mehr als 10 Mal / Tag
- ☐ Mehrmals in der Woche
- ☐ Mehrmals im Monat
- ☐ Ich spiele nicht
- ☐ Weiß nicht

13. In einem digitalen Spiel zum Thema "Ernährung" sollen Ziele gemeinsam in einem Team erreicht werden. Wer sollten die Spieler in diesem Team sein?
(Kreuze bitte alle passenden Antworten an!)

- ☐ Familienmitglieder
- ☐ Freunde/innen
- ☐ Personen mit gleichen Hobbies
- ☐ Personen mit gleichen Essgewohnheiten
- ☐ Personen ohne Gemeinsamkeiten
- ☐ Anderes Team:________________________
- ☐ Kein Team

14. Mit wie vielen Spielern (im Spiel) spielst Du am liebsten gleichzeitig digitale Spiele?
(Kreuze bitte nur eine Antwort an!)

- ☐ Mit 2-5 Spielern
- ☐ Mit 6-10 Spielern
- ☐ Mit mehr als 10 Spielern
- ☐ Alleine
- ☐ Ich spiele nicht
- ☐ Weiß nicht

15. Welche Spielfigur würde Dir in einem digitalen Spiel am besten gefallen?
(Kreuze bitte nur eine Antwort an!)

- ☐ Niedliches Tier (Kätzchen)
- ☐ Beeindruckendes Tier (Raubkatze)
- ☐ Fantasiefigur (Held, Ritter, Fee)
- ☐ Fantasietier (Furby, Tamagotchi)
- ☐ Mensch, der genauso alt ist wie ich
- ☐ Mensch, der älter ist als ich
- ☐ Andere Spielfigur:________________________

16. Möchtest Du in einem digitalen Spiel Informationen über Ernährung erhalten?
(Kreuze bitte nur eine Antwort an!)

- ☐ Ja
- ☐ Nein

17. Auf welche Art und Weise würdest Du in einem digitalen Spiel etwas über Ernährung lernen wollen?
(Kreuze bitte alle passenden Antworten an!)

- ☐ Durch Quizfragen
- ☐ Durch Lösen von Aufgaben
- ☐ Durch lustige Filme
- ☐ Durch Mitspieler/innen
- ☐ Andere Art:________________________
- ☐ Ich möchte nichts lernen
- ☐ Keine Antwort trifft zu
