# Supplementary material for: Digital Gaming for Nutritional Education: A Survey on Preferences, Motives, and Needs of Children and Adolescents
Source: JMIR Form Res. 2019 Feb 13;3(1):e10284. doi: 10.2196/10284 (PMC6391648; doi:10.2196/10284)
Supplement: Multimedia Appendix 2 [file formative_v3i1e10284_app2.pdf]

Multimedia Appendix 2. Digital gameplay: motives, behavior, and preferences

Digital gaming		All n (%)	Girls n (%)	Boys n (%)
**Motives**[a;1]		282 (100)		
Emotion				
	Pleasure	209 (74.1)	78 (59.5)	131 (86.8)
	Happiness	24 (8.5)	4 (3.1)	20 (13.3)
	Sadness	15 (5.3)	7 (5.3)	8 (5.3)
	Boredom	164 (58.2)	78 (59.5)	86 (57.0)
Setting				
	Friends	54 (19.2)	16 (12.2)	38 (25.2)
	School	13 (4.6)	3 (2.3)	10 (6.6)
	On the go	48 (17.0)	26 (19.9)	22 (14.6)
	Alone (at home)	117 (41.5)	47 (35.9)	70 (46.4)
Other		9 (3.2)	1 (0.8)	8 (5.3)
I don’t play		29 (10.3)	22 (16.8)	7 (4.6)
I don’t know		3 (1.1)	2 (1.5)	1 (0.7)
**Behavior**				
Digital game[b;2]		283 (100)		
	Yes	242 (85.5)	104 (78.8)	138 (91.4)
	No	41 (14.5)	28 (21.2)	13 (8.6)
Duration (smartphone, tablet)[a;3]		227 (100)		
	≤ 30 minute	151 (66.5)	64 (58.2)	87 (74.4)
	=60 minute	24 (10.6)	12 (10.9)	12 (10.3)
	>60 minute	17 (7.5)	10 (9.1)	7 (6.0)
	I do not play	33 (14.5)	24 (21.8)	9 (7.7)
	I do not know	2 (0.9)	0 (0.0)	2 (1.7)
Duration (PC, console)[a;4]		209 (100)		
	≤ 30 minute	57 (27.3)	34 (43.0)	23 (17.7)
	=60 minute	63 (30.1)	14 (17.7)	49 (37.7)
	>60 minute	53 (25.4)	6 (7.6)	47 (36.2)
	I do not play	35 (16.8)	25 (31.7)	10 (7.7)
	I do not know	1 (0.5)	0 (0.00)	1 (0.8)

Frequency (smartphone, tablet)[a;5]		236 (100)		
	Daily	144 (61.0)	60 (51.7)	84 (70.0)
	Weekly	27 (11.4)	17 (14.7)	10 (8.3)
	Monthly	31 (13.1)	14 (12.1)	17 (14.2)
	I do not play	31 (13.1)	23 (19.8)	8 (6.7)
	I do not know	3 (1.3)	2 (1.7)	1 (0.8)
Frequency (PC, console)[a;6]		204 (100)		
	Daily	82 (40.2)	21 (28.4)	61 (46.9)
	Weekly	47 (23.0)	12 (16.2)	35 (26.9)
	Monthly	39 (19.1)	14 (18.9)	25 (19.2)
	I do not play	32 (15.7)	24 (32.4)	8 (6.2)
	I do not know	4 (2.0)	3 (4.1)	1 (0.8)
**Preferences**				
Team composition[b;7]		286 (100)		
	Family	78 (27.3)	40 (29.4)	38 (25.3)
	Friends	191 (66.8)	87 (64.0)	104 (69.3)
	Same hobbies	97 (33.9)	41 (30.2)	56 (37.3)
	Same eating behavior	106 (37.1)	57 (41.9)	49 (32.7)
	No similarities	37 (12.9)	16 (11.8)	21 (14.0)
	Other	9 (3.2)	4 (2.9)	5 (3.3)
	No team	21 (7.4)	11 (8.1)	10 (6.7)
Team mates[b;8]		255 (100)		
	≤ 5	116 (45.5)	38 (34.2)	78 (54.2)
	6-10	23 (9.0)	4 (3.6)	19 (13.2)
	>10	27 (10.6)	7 (6.3)	20 (13.9)
	Alone	58 (22.8)	40 (36.0)	18 (12.5)
	I do not play	28 (11.0)	20 (18.0)	8 (5.6)
	I do not know	3 (1.2)	2 (1.8)	1 (0.7)
Game character[a;9]		277 (100)		
	Cute animal	16 (5.8)	6 (4.7)	10 (6.7)
	Impressive animal	28 (10.1)	14 (10.9)	14 (9.4)
	Fantasy animal	14 (5.1)	8 (6.3)	6 (4.0)
	Fantasy character	53 (19.1)	27 (21.1)	26 (17.5)
	Human being	119 (43.0)	53 (41.4)	66 (44.3)
	Other	21 (7.6)	8 (6.3)	13 (8.7)
	No answer applies.	26 (9.4)	12 (9.4)	14 (9.4)

**Nutritional game**				
Information[a;10]		280 (100)		
	Yes	144 (51.4)	82 (62.1)	62 (41.9)
	No	136 (48.6)	50 (37.9)	86 (58.1)
Learning[b;11]		287 (100)		
	Quiz	157 (54.7)	94 (69.1)	63 (41.7)
	Task	123 (42.9)	74 (54.4)	49 (32.5)
	Movies	66 (23.0)	33 (24.3)	33 (21.9)
	Mates	55 (19.2)	24 (17.7)	31 (20.5)
	Other	9 (3.1)	1 (0.7)	8 (5.3)
	No learning	37 (12.9)	8 (5.9)	29 (19.2)
	No answer applies	16 (5.6)	5 (3.7)	11 (7.3)

PC, personal computer; [a]Single response allowed. [b]Multiple responses allowed.

[1]When do you often play digital games?

[2]Do you play digital games (smartphone, PC, console, apps)?

[3]How long do you play digital games on your smartphone/tablet/apps without any interruption?

[4]How long do you play digital games on your PC/console without any interruption?

[5]How often do you play digital games on your smartphone/tablet/apps?

[6]How often do you play digital games on your PC/console?

[7]In a digital game about “nutrition,” goals are to be achieved together in a team. Who should the players on this team be?

[8]With how many players (in the game) do you like to play digital games at the same time?

[9]Which character would you like best in a digital game?

[10]Do you like to receive nutrition information in a digital game?

[11]How would you like to learn about nutrition in a digital game?
